# Supplementary material for: MetaboNet-Bench: A Multi-modal Benchmark for Glucose Forecasting in Type 1 Diabetes
Source: ArXiv. 2026 Jun 25:arXiv:2606.18640v2. Preprint. [Version 2] (PMC13321326)
Supplement: Supplement 1 [file NIHPP2606.18640v2-supplement-1.pdf]

A Appendix - Datasets

Table 3: Overview of all datasets included in the public release of the MetaboNet dataset (Wolff et al., 2026), showing the number of subjects per dataset. The column "# Not MDI" reports the number of subjects using continuous insulin pump therapy, i.e., not treated with Multiple Daily Injections (MDI). The MetaboNet consolidated dataset is distributed under a custom license that restricts use to non-commercial research and educational purposes. Component datasets retain their original licenses, with studies hosted by the Jaeb Center for Health Research governed by the terms of use (JAEB ToU) and the remaining datasets released under the Creative Commons Attribution 4.0 International license (CC BY 4.0).

Dataset Name	Citation	# Subjects	# Not MDI	Carbs Available	License
CTR3	Zisser et al. (2014)	30	30	✓	JAEB ToU
DCLP3	Brown et al. (2019)	112	112	×	JAEB ToU
DCLP5	Breton et al. (2020)	100	100	×	JAEB ToU
Flair	Bergenstal et al. (2021)	113	113	×	JAEB ToU
IOBP2	Lynch et al. (2022)	332	332	×	JAEB ToU
Loop Observational Study	Lum et al. (2021)	845	845	✓	JAEB ToU
PEDAP	Wadwa et al. (2023)	65	65	✓	JAEB ToU
ReplaceBG	Aleppo et al. (2017)	208	208	✓	JAEB ToU
AZT1D	Khamesian et al. (2025)	23	23	✓	CC BY 4.0
BrisT1D	Gordon et al. (2025)	19	19	✓	CC BY 4.0
HUPA-UCM	Hidalgo et al. (2024)	22	12	✓	CC BY 4.0
Shanghai T1DM	Zhao et al. (2023)	12	9	✓	CC BY 4.0
T1D-UOM	Alsuhaymi et al. (2025)	14	6	✓	CC BY 4.0
**Total**		**1895**	**1874**		

B Appendix — Implementation Details

B.1 Training details and hyperparameters

Gluformer: Gluformer was trained following instructions on the GitHub (Sergazinov et al., 2022). Gluformer was trained using the Adam optimizer with a learning rate of 0.001 for 50 epochs with negative log-likelihood loss over a mixture distribution of future glucose trajectories. These parameters were selected based on recommended defaults in the paper and associated open source code. Training uses contiguous 16-hour sequences from the dataset, with 15 hours of input and 1 hour of target values. We used the log sum likelihood loss described in Sergazinov et al. (2024). To match the paper, we generated 5-dimensional time embeddings for each input and predicted sample. The time embedding consists of day of year, day of month, day of week, hour of day and minute of hour, each normalized to [0, 1].

LightGBM: For LightGBM, sequences from the MetaboNet training set were converted into a flat tabular representation with two hours of time-lagged features (history length $H = 24$ at the dataset's 5-minute sampling rate), with windows generated using non-overlapping episodes. The prediction horizons were fit jointly via scikit-learn's `MultiOutputRegressor`, which trains one LightGBM regressor per horizon, tuned with 100 trials of Optuna using the default Tree-structured Parzen Estimator (TPE) sampler over a custom-defined search space. For each trial the sampler proposed a configuration consisting of the number of boosting rounds $n_{\text{estimators}} \in [50, 500]$, the maximum tree depth $\in [3, 12]$, the learning rate $\in [0.01, 0.3]$ on a logarithmic scale, the maximum number of leaves per tree $\in [10, 300]$, the per-tree feature subsampling fraction $\in [0.5, 1.0]$, the per-tree row subsampling (bagging) fraction $\in [0.5, 1.0]$, the bagging frequency $\in [0, 7]$ iterations, the minimum number of samples per leaf $\in [5, 100]$, and the L_1 and L_2 leaf-weight regularization strengths $\lambda_1, \lambda_2 \in [0, 10]$. Each candidate was scored by the mean root mean squared error (RMSE) under 3-fold cross-validation on the training split. The configuration achieving the lowest mean CV-RMSE was retained and refit on the full training split before evaluation. The random seed was fixed to 42.

The Optuna-selected configurations differ across ablations. With the full feature set (CGM + Carbs + Insulin), the best configuration uses 474 boosting rounds at depth 10 with 277 leaves, learning rate 0.079, feature fraction 0.78, bagging fraction 0.59 at frequency 0, 38 samples per leaf, and regularization $\lambda_1 = 6.73$, $\lambda_2 = 7.83$. The CGM + Carbs ablation uses 480 rounds at depth 12

with 294 leaves, learning rate 0.060, feature fraction 0.90, bagging fraction 0.81 at frequency 2, 95 samples per leaf, and $\lambda_1 = 6.40$, $\lambda_2 = 8.98$. The CGM-only ablation uses 388 rounds at depth 12 with 284 leaves, learning rate 0.066, feature fraction 0.86, bagging fraction 0.98 at frequency 3, 64 samples per leaf, and $\lambda_1 = 8.27$, $\lambda_2 = 2.45$. Finally, the CGM + Insulin ablation uses 454 rounds at depth 11 with 239 leaves, learning rate 0.084, feature fraction 0.78, bagging fraction 0.72 at frequency 0, 91 samples per leaf, and $\lambda_1 = 0.22$, $\lambda_2 = 2.28$.

UniTS: The UniTS model was prompt-tuned using the Adam optimizer with a batch size of 256 and learning rate and weight decay recommended by Optuna. We used 30 trials of Optuna using the default TPE sampler over learning rates $\in [0.00001, 0.001]$ and weight decays $\in [0.00001, 0.001]$. Each ablation was tuned separately. With the full feature set (CGM + Insulin + Carbs) the best configuration uses a learning rate of 0.00087 and a weight decay of 0.0000102. The CGM + Insulin ablation uses a learning rate of 0.00093 and weight decay of 0.0000105. The CGM + carbs ablation uses a learning rate of 0.00065 and weight decay of 0.00033. The CGM only ablation uses a learning rate of 0.00087 and weight decay of 0.0000102.

LSTM: The LSTM models were trained using the Adam optimizer with MSE loss. We use the tanh activation function for cell state updates and the sigmoid activation function for input, forget and output gates. Hyperparameters and their values were tuned identically to UniTS. With the full feature set (CGM + Insulin + Carbs) the best configuration uses a learning rate of 0.00098 and a weight decay of 0.0000109. The CGM + Insulin ablation uses a learning rate of 0.001 and weight decay of 0.00001. The CGM + carbs ablation uses a learning rate of 0.00096 and weight decay of 0.000012. The CGM only ablation uses a learning rate of 0.00087 and weight decay of 0.0000102.

GluForecast: The GluForecast models were trained using the Adam optimizer with MSE loss. The model uses an inner dimension of 128 with 4 heads, 4 transformer layers and a dropout of 0.1 between each layer. Hyperparamters and their values were tuned identically to UniTS and LSTM. With the full feature set (CGM + Insulin + Carbs) the best configuration uses a learning rate of 0.00087 and a weight decay of 0.0000108. The CGM + Insulin ablation uses a learning rate of 0.00087 and weight decay of 0.000088. The CGM + carbs ablation uses a learning rate of 0.00092 and weight decay of 0.000049. The CGM only ablation uses a learning rate of 0.00048 and weight decay of 0.000013.

Ridge: Ridge was trained with an identical preprocessing and hyperparameter tuning setup as LightGBM, with two differences: features were standardized to zero mean and unit variance via a scikit-learn `Pipeline` with `StandardScaler` before fitting, and the search space reduced to the single regularization strength $\alpha \in [0.1, 100]$ on a logarithmic scale. The Optuna-selected α was 1.378 for the full feature set (CGM + Carbs + Insulin), 0.523 for the CGM + Carbs ablation, 1.377 for the CGM-only ablation, and 1.358 for the CGM + Insulin ablation.

B.2 GluForecast

We generated time embeddings with 4 dimensions as follows:

Let t denote a timestamp measured in seconds. We define the day and week periods as:

$$D = 24 \cdot 60 \cdot 60, \qquad W = 7D$$

$$t_{day} = t \bmod D \qquad t_{week} = t \bmod W$$

The angular phases are given by

$$\theta_{\text{day}}(t) = 2\pi \frac{t_{day}}{D}, \qquad \theta_{\text{week}}(t) = 2\pi \frac{t_{week}}{W}.$$

The time embedding is then defined as

$$\phi(t) = \begin{bmatrix} \sin(\theta_{\text{day}}(t)) \\ \cos(\theta_{\text{day}}(t)) \\ \sin(\theta_{\text{week}}(t)) \\ \cos(\theta_{\text{week}}(t)) \end{bmatrix}$$

This time embedding unambiguously expresses time in cyclical day and week patterns, giving the model the best chance to learn daily and weekly patterns affecting glucose.

During training, loss is calculated as follows:

$$\mathcal{L} = \sum_{h=1}^{H} w_h \sum_{t=c}^{T-h} \ell(y_t + \hat{y}_{h,t},\, y_{t+h}), \qquad w_h = 10 + h.$$

Horizon weight: We chose to weigh longer horizons more heavily to nudge the model to prioritize the more difficult prediction task. The constant 10 balances the emphasis on long and short tasks. The moderately large horizon weights counteract the small deltas generated by the model.

Variables: H is the number of prediction heads (horizons); $h \in \{1, \ldots, H\}$ indexes the horizon (in 5 minute increments); t indexes time (in 5 minute increments); c is the first time index included in the loss to allow a minimum context; T is the final time index of the sequence; y_t is the ground-truth glucose value at time t; $\hat{y}_{h,t}$ is the model predicted change in glucose for horizon h made at time t; $\ell(\cdot, \cdot)$ is the per-timestep loss; and w_h is the horizon-dependent weight applied to head h.

B.3 Model Input

Table 4: Model inputs

Model	Timestamps	CGM	Insulin	Carbs	Input length
GluForecast	X	X	X	X	167
Gluformer	X	X			180
LE		X			1
LightGBM	X	X	X	X	24
LSTM		X	X	X	180
Ridge	X	X	X	X	24
UniTS		X	X	X	180
ZOH		X			1

B.4 Model Complexity

Table 5 shows the complexity of each model.

Model	Params	FLOPs	MACs
Gluformer	11.2M	2.727G	1.308G
UniTS	2.69M	188.93M	94.2M
GluForecast	819K	264.78M	131.74M
LSTM	202K	71.8M	1.54K
Ridge	1048	-	-
LightGBM	nonparametric	-	-

Table 5: This table summarizes the model sizes and their runtime complexity, measured in Floating Point Operations (FLOPS) and Multiply-Accumulate Operations (MACs).

B.5 Implementation Details and Hardware

All models were trained on a single H200 GPU with 141GB of VRAM unless otherwise mentioned. Each model was trained until evaluation loss plateaued. UniTS took about two hours to train for 20 epochs. LSTM took about one hour to train for 20 epochs. GluForecast took about 4 hours to train for 40 epochs. Other models were less computation resource-intensive and were trained on a CPU node with AMD EPYC 7301 16-Core Processor with 503GB memory. Full end to end evaluation including benchmarking, metrics calculation and figure generation takes about one hour on H200. The evaluation did not focus on execution efficiency for model training and testing. Thus, detailed information regarding memory consumption and time cost was not collected for reporting purposes.

C Appendix - Metrics

Conventional Metrics: We selected RMSE and MARD because of their popularity for forecasting models. We reported model size as it dictates which systems will be capable of running each model, as well as FLOPS to indicate the computation complexity of each model and multiply-accumulate operations (MACs) to indicate how much common optimization libraries and dedicated acceleration hardware might speed up inference.

RMSE and MARD are calculated as follows:

$$RMSE = \sqrt{\frac{1}{n}\sum_{i=1}^{n}(y_i - \hat{y}_i)^2}$$

$$MARD = \frac{1}{n}\sum_{1}^{n}|y_i - \hat{y}_i|$$

where n is the number of samples, y_i is the reference value at location i and $\hat{y}_i$ is the prediction at location i.

Clinical Metrics: The DTS error grid indicates the clinical significance or seriousness of model error (Klonoff et al., 2024). It is an industry-standard visualization that maps each prediction-reference pair to a clinically-defined risk zone based on the real-world implications of the error. The zone counts for the DTS error grid indicate how many of the predictions fall within each clinical zone - with zone A representing clinically safe predictions and zone B through E indicating errors with increasingly serious clinical consequences. Compared with alternate error grids (e.g., Clarke), the DTS framework provides a modern, expert-defined, and treatment-relevant characterization of risk. In our results, we report the percentage of time spent in each error zone, grouping C, D, and E together for reporting simplicity.

We used the following definitions of *postprandial* and *correction bolus* in order to produce figures in figures 4, 10, 9, and 5.

Postprandial: For a given sample, we look at the current reported carbohydrates as well as the reported carbs every 5 minutes up until 30 minutes prior to the sample. If any of these carbs are nonzero we say the sample is *postprandial*.

Correction Bolus: If a sample has a CGM value > 250 mg/dL and an insulin value of > 2IU for any of the intervals until 30 minutes prior to the sample, we call that sample a *correction bolus*.

D Appendix — Supplementary Tables

Table 6: Performance comparison of glucose prediction models across different prediction horizons. The best (lowest) values for each horizon are highlighted in bold.

**RMSE (mg/dL)**												
PH (min)	5	10	15	20	25	30	35	40	45	50	55	60
GluForecast	**3.57**	**6.85**	**10.39**	**13.83**	**17.03**	**19.95**	**22.65**	**25.13**	**27.40**	**29.50**	**31.41**	**33.17**
Gluformer	5.28	8.24	11.86	15.38	18.82	21.97	24.87	27.53	29.94	32.11	34.13	36.35
LightGBM	3.69	7.18	10.89	14.47	17.79	20.84	23.64	26.21	28.56	30.72	32.69	34.48
LE	4.68	9.50	14.69	19.99	25.31	30.60	35.84	41.02	46.14	51.17	56.10	60.92
LSTM	4.27	7.01	10.49	13.89	17.11	20.06	22.78	25.29	27.57	29.69	31.64	33.41
Ridge	3.85	7.56	11.46	15.24	18.77	22.03	25.03	27.77	30.27	32.54	34.60	36.46
UniTS	4.01	7.05	10.60	14.10	17.42	20.44	23.26	25.79	28.18	30.33	32.37	34.14
ZOH	5.71	10.62	15.11	19.19	22.90	26.28	29.37	32.22	34.84	37.25	39.47	41.52
**MARD (%)**												
PH (min)	5	10	15	20	25	30	35	40	45	50	55	60
GluForecast	**1.69**	**3.47**	**5.42**	**7.27**	9.11	**10.76**	**12.33**	**13.84**	15.23	16.57	17.78	18.91
Gluformer	2.60	4.10	6.09	8.06	9.99	11.69	13.31	14.73	16.03	17.32	18.52	19.57
LightGBM	1.76	3.67	5.71	7.68	9.54	11.27	12.90	14.43	15.85	17.18	18.41	19.55
LE	2.32	4.83	7.47	10.15	12.84	15.54	18.25	20.94	23.62	26.27	28.90	31.50
LSTM	2.31	3.72	5.57	7.45	**9.11**	10.89	12.40	**13.81**	**15.06**	**16.39**	**17.54**	**18.58**
Ridge	1.79	3.81	5.97	8.06	10.05	11.94	13.71	15.37	16.91	18.33	19.65	20.86
UniTS	1.97	3.62	5.56	7.43	9.34	10.93	12.49	13.91	15.37	16.49	17.73	18.79
ZOH	2.91	5.49	7.85	10.00	11.98	13.80	15.49	17.06	18.51	19.86	21.12	22.29

Table 7: Performance comparison on the **Task 1: Novel Patients split** of glucose prediction models across different horizons and metrics. The best (lowest) values for each horizon are highlighted in bold. This captures how well models generalize to new patients.

**RMSE (mg/dL)**												
PH (min)	5	10	15	20	25	30	35	40	45	50	55	60
GluForecast	**3.58**	**6.88**	**10.46**	**13.91**	**17.13**	**20.06**	**22.76**	**25.23**	**27.50**	**29.59**	**31.49**	**33.23**
Gluformer	5.30	8.24	11.91	15.45	18.91	22.05	24.96	27.60	30.00	32.16	34.17	36.39
LightGBM	3.71	7.22	10.96	14.55	17.89	20.95	23.75	26.31	28.65	30.79	32.75	34.51
LE	4.72	9.58	14.83	20.18	25.56	30.89	36.17	41.38	46.52	51.57	56.52	61.36
LSTM	4.29	7.04	10.55	13.97	17.20	20.16	22.88	25.38	27.65	29.75	31.69	33.45
Ridge	3.87	7.60	11.54	15.34	18.89	22.16	25.15	27.89	30.37	32.63	34.67	36.51
UniTS	4.02	7.08	10.66	14.19	17.52	20.55	23.38	25.91	28.29	30.43	32.45	34.20
ZOH	5.74	10.69	15.22	19.31	23.04	26.42	29.52	32.35	34.96	37.35	39.56	41.60
**MARD (%)**												
PH (min)	5	10	15	20	25	30	35	40	45	50	55	60
GluForecast	**1.70**	**3.49**	**5.47**	**7.35**	9.22	**10.90**	**12.50**	14.03	15.44	16.80	18.02	19.17
Gluformer	2.62	4.13	6.15	8.15	10.11	11.83	13.48	14.92	16.24	17.55	18.75	19.82
LightGBM	1.77	3.70	5.77	7.77	9.66	11.43	13.09	14.65	16.09	17.43	18.68	19.83
LE	2.34	4.89	7.57	10.30	13.03	15.77	18.52	21.25	23.96	26.65	29.30	31.93
LSTM	2.32	3.76	5.63	7.53	**9.21**	11.03	12.56	**13.99**	**15.25**	**16.59**	**17.75**	**18.80**
Ridge	1.80	3.85	6.03	8.16	10.18	12.09	13.89	15.58	17.13	18.56	19.89	21.10
UniTS	1.97	3.65	5.62	7.51	9.44	11.06	12.64	14.08	15.56	16.68	17.93	18.99
ZOH	2.94	5.56	7.96	10.14	12.14	13.98	15.70	17.28	18.75	20.10	21.36	22.54

Table 8: Performance comparison on the **Task 2: Known Patients split** dataset of glucose prediction models across different horizons and metrics. This captures how well the models perform when they are trained on earlier data from the same patient they are tested on. The best (lowest) values for each horizon are highlighted in bold.

**RMSE (mg/dL)**												
PH (min)	5	10	15	20	25	30	35	40	45	50	55	60
GluForecast	**3.52**	**6.77**	**10.24**	**13.64**	**16.79**	**19.70**	**22.38**	**24.87**	**27.16**	**29.29**	**31.24**	**33.03**
Gluformer	5.25	8.23	11.74	15.21	18.62	21.76	24.67	27.35	29.79	31.99	34.04	36.26
LightGBM	3.65	7.10	10.74	14.28	17.56	20.59	23.39	25.98	28.34	30.54	32.56	34.39
LE	4.59	9.31	14.36	19.54	24.73	29.92	35.08	40.18	45.23	50.22	55.11	59.89
LSTM	4.24	6.93	10.36	13.72	16.90	19.82	22.55	25.07	27.37	29.54	31.53	33.33
Ridge	3.79	7.46	11.28	15.01	18.50	21.74	24.72	27.48	30.01	32.34	34.45	36.36
UniTS	3.97	6.99	10.46	13.91	17.18	20.17	22.98	25.52	27.93	30.12	32.17	34.00
ZOH	5.62	10.46	14.87	18.89	22.57	25.94	29.03	31.90	34.54	37.00	39.26	41.34
**MARD (%)**												
PH (min)	5	10	15	20	25	30	35	40	45	50	55	60
GluForecast	**1.67**	**3.40**	**5.29**	**7.08**	**8.85**	**10.44**	**11.93**	**13.38**	14.72	16.04	17.21	18.31
Gluformer	2.55	4.02	5.94	7.84	9.70	11.36	12.92	14.28	15.53	16.80	17.98	18.99
LightGBM	1.73	3.59	5.56	7.46	9.25	10.91	12.47	13.94	15.30	16.60	17.80	18.91
LE	2.27	4.69	7.24	9.82	12.41	15.01	17.62	20.22	22.81	25.41	27.97	30.49
LSTM	2.28	3.65	5.44	7.25	8.86	10.57	12.03	13.39	**14.61**	**15.91**	**17.05**	**18.08**
Ridge	1.76	3.73	5.81	7.84	9.76	11.58	13.29	14.89	16.39	17.80	19.09	20.29
UniTS	1.95	3.56	5.44	7.25	9.10	10.63	12.14	13.52	14.93	16.04	17.27	18.32
ZOH	2.83	5.32	7.60	9.68	11.60	13.37	15.01	16.54	17.96	19.30	20.54	21.70

E Appendix — Supplementary Figures

E.1 Performance on Standard Metrics

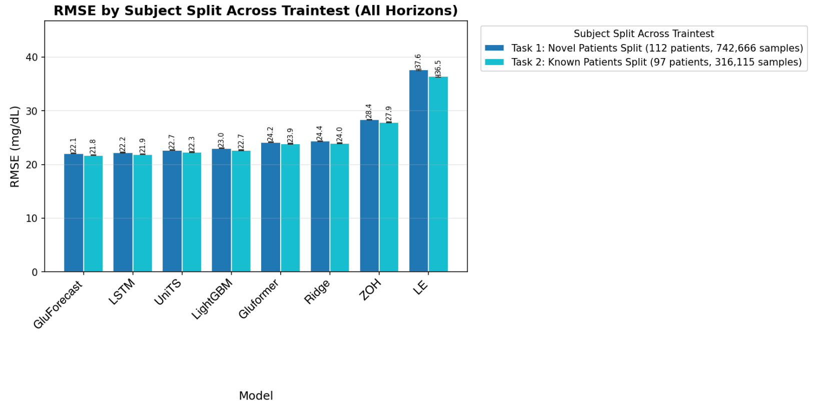

Figure 6: RMSE for Novel Patients, vs Known Patients with some data in the training set. The RMSE is calculated across all prediction horizons. The MetaboNet dataset comes with a predefined train/test split. In order to allow for testing generalization to new patients, within the test set, a portion of patients have their data split between train and test (known patients), and a portion do not (novel patients) Wolff et al. (2026). This figure shows that exposure to patients during training provides very minimal but consistent performance gains. Unless otherwise specified we draw from the *full* test set in all other evaluations.

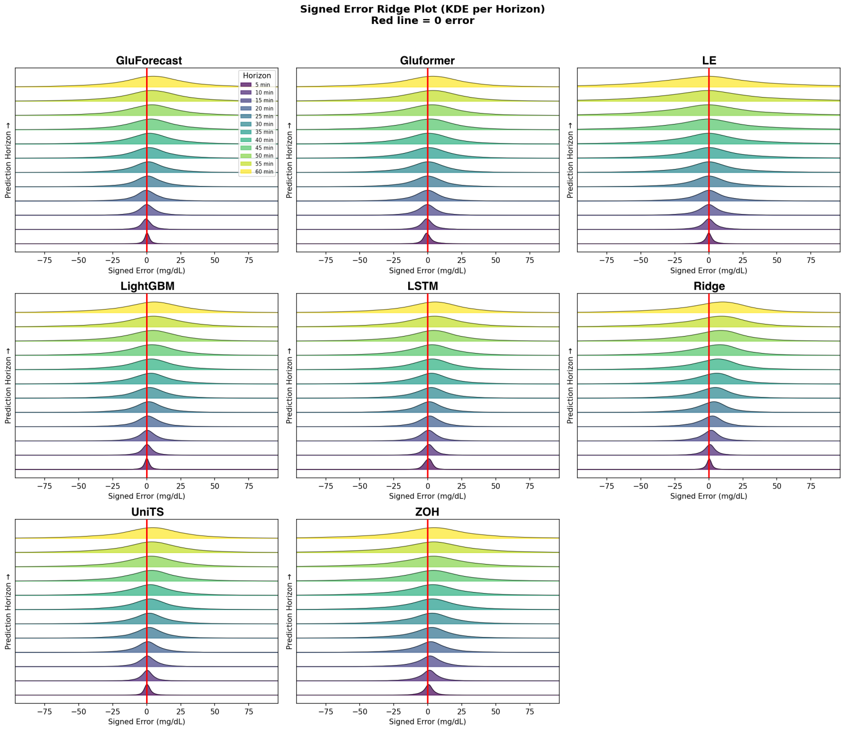

Figure 7: Illustration of the signed error for each model across prediction horizons, from shorter horizons at the bottom to longer horizons at the top. The Kernel Density Error (KDE) is visualized for each prediction horizon.

E.2 Evaluation Across Glycemic Profiles

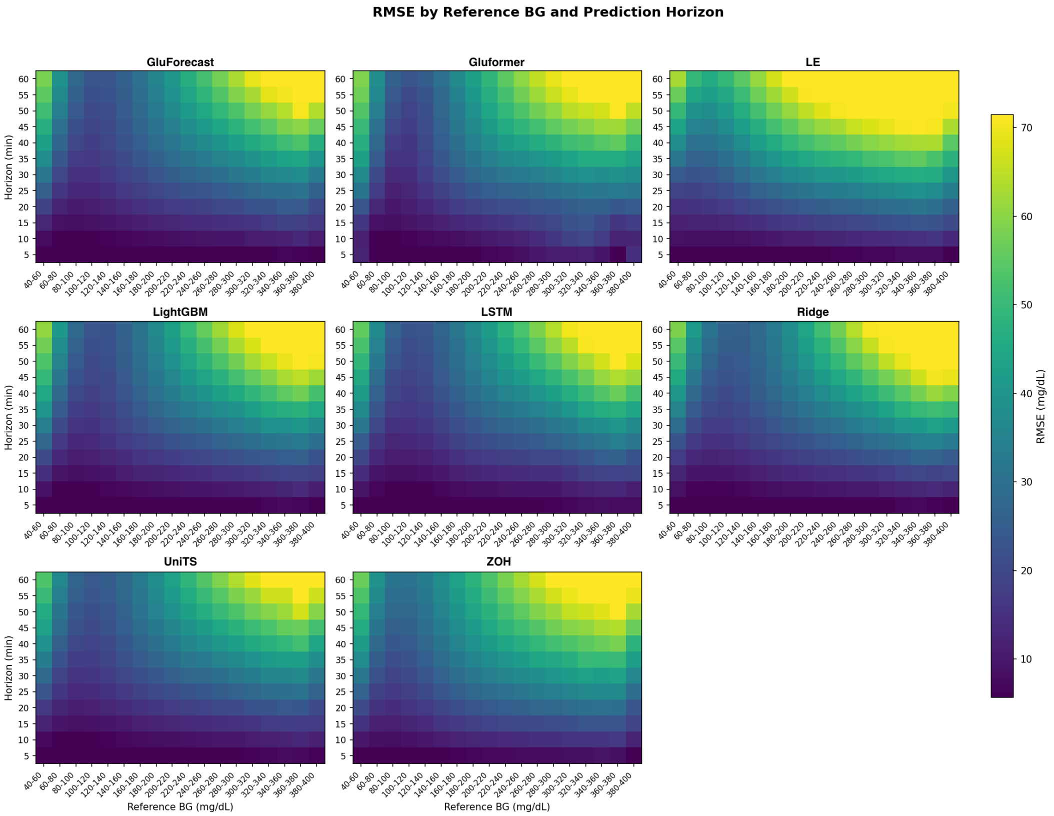

Figure 8: Heatmap illustrating, for each model, the relationship between prediction horizon, reference blood glucose (BG) values, and RMSE. This illustrates which blood glucose ranges the models have trouble predicting and at which time horizons

E.3 Multimodal Performance at Mealtime and Correction

We performed some analysis comparing model performance during particularly clinically difficult situations, namely, meals, and hyperglycemic correction insulin. This analysis shows that the multimodal GluForecast model performs notably better for post-prandial predictions (see Figure 9). The multimodal GluForecast also performs better than otherwise following corrections (Figure 10).

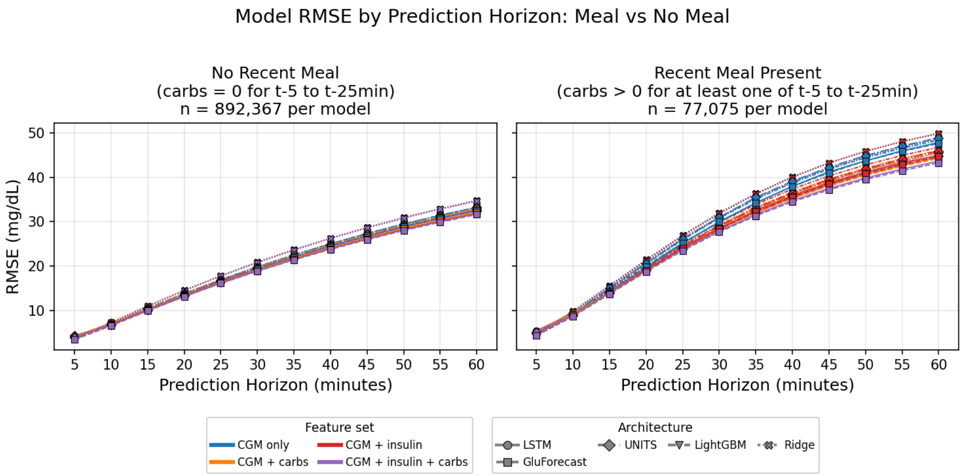

Figure 9: Comparison of model RMSE when there are no recent meals present in the data (left), compared to when there was a meal present during the previous 30 minutes (right).

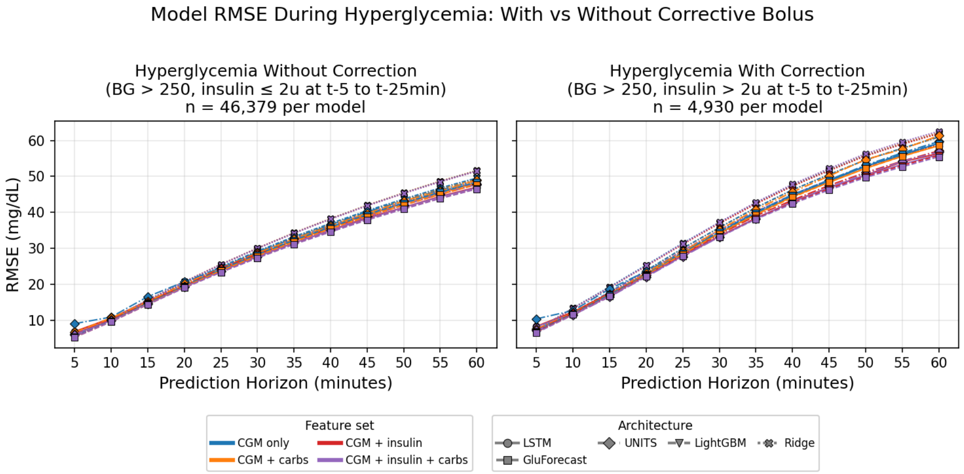

Figure 10: Comparison of model RMSE during hyperglycemic samples in the true values of the dataset. The left plot shows model performance when no correction insulin is given, while the right plot shows examples for when a dose larger than 2U of insulin is given within the previous 30 minutes.

E.4 Evaluation Across Subpopulations

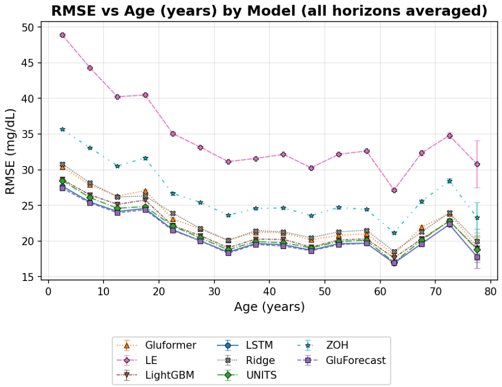

Age

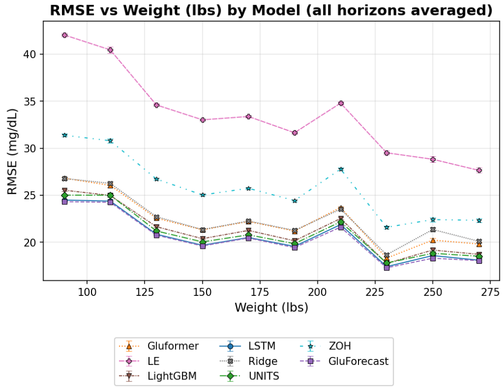

Weight

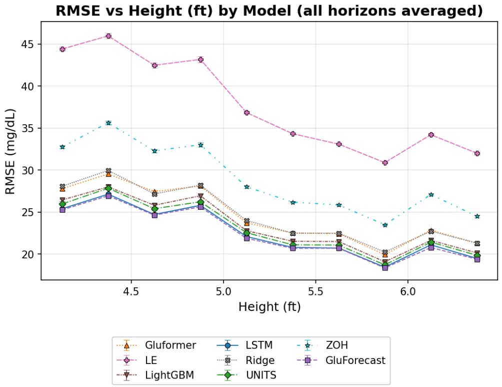

Height

Figure 11: RMSE results across subpopulations aggregated over all prediction horizons, illustrating the impact of age, weight, and height on model performance.

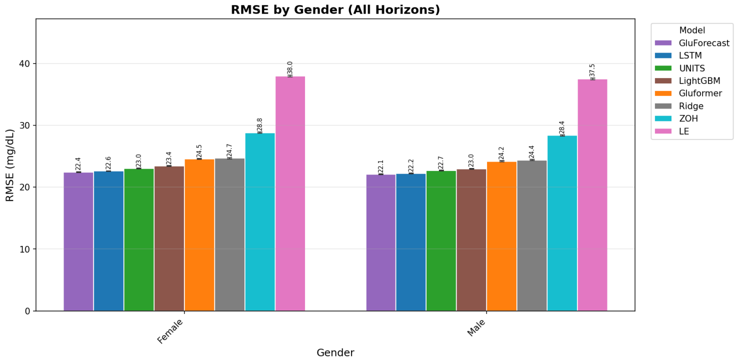

Figure 12: RMSE results across subpopulations aggregated over all prediction horizons, illustrating the impact of gender on model performance.
